# Immunocyte phenotype and breast cancer risk: A Mendel randomization analysis

**DOI:** 10.1371/journal.pone.0311172

**Published:** 2024-10-17

**Authors:** Bolin Li, Xinmeng Li, Jialing Liu, Yuanhe Gao, Yan Li

**Affiliations:** 1 The Graduate School, Heilongjiang University of Chinese Medicine, Harbin, China; 2 First Affiliated Hospital, Heilongjiang University of Chinese Medicine, Harbin, China; Gangnam Severance Hospital, Yonsei University College of Medicine, REPUBLIC OF KOREA

## Abstract

**Background:**

Breast cancer remains a significant global health challenge. Understanding its etiological factors, particularly the role of immune system components, is crucial. This study leverages Mendelian randomization (MR) to investigate the causal relationship between various immune cell features and the risk of developing breast cancer.

**Methods:**

Utilizing two-sample MR analysis, we examined 731 immune cell features across 7 groups for their potential causal links to breast cancer. We analyzed genome-wide association studies (GWAS) data of 257,730 Europeans, comprising 17,389 cases and 240,341 controls, focusing on 24,133,589 single nucleotide polymorphisms (SNPs). Instrumental variables (IVs) were selected based on genetic associations, with rigorous statistical methods employed, including inverse variance weighting (IVW) and weighted median-based estimation.

**Results:**

Our analysis identified 20 immunophenotypes with significant causal associations with breast cancer risk. Notably, contain B cell, mature T cell, T + B + NK (TBNK) cells, regulatory T (Treg) cell, Classic dendritic cells (cDCs), Monocyte, and Myeloid cell group features displayed positive or negative correlations with breast cancer. For instance, specific B cell phenotypes were found to have both positive and negative causal relationships with breast cancer. Additionally, reverse MR analysis revealed no significant causal effects of breast cancer on these immune characteristics.

**Conclusions:**

This study underscores the complex interplay between various immune cell phenotypes and breast cancer risk. The identified immunophenotypes could be potential biomarkers or targets for future therapeutic interventions. Our findings contribute to a deeper understanding of the immunological dimensions of breast cancer etiology.

## Introduction

Breast cancer, a leading cause of cancer-related morbidity and mortality among women worldwide, presents a substantial clinical challenge due to its complex etiology and diverse phenotypic presentations [1]. Despite advances in understanding the genetic and environmental factors contributing to breast cancer, the intricate interplay between these factors and the immune system’s role remains less explored [2]. The immune system, known for its dual role in both promoting and inhibiting tumorigenesis, has emerged as a pivotal player in the development and progression of breast cancer [3]. This study focuses on elucidating the causal relationships between specific immune cell phenotypes and the risk of breast cancer, leveraging the robust framework of Mendelian randomization (MR) to overcome the limitations of observational studies. Recent research has increasingly recognized the significance of the immune system in cancer biology [4, 5]. Immune cells in the tumor microenvironment can either suppress tumor growth by recognizing and eliminating cancer cells or facilitate tumorigenesis by creating an immunosuppressive network that promotes tumor survival and metastasis [6]. This paradoxical role makes the study of immune components in cancer particularly challenging yet crucial for developing targeted therapies [7]. The advent of genome-wide association studies (GWAS) has revolutionized our understanding of breast cancer genetics [8]. GWAS have identified numerous genetic loci associated with breast cancer risk, but translating these findings into actionable clinical insights has been hampered by the complexity of genetic-phenotypic correlations [9]. Mendelian randomization, a method that uses genetic variants as instrumental variables (IVs) to infer causal relationships between exposures and outcomes, offers a solution to this challenge [10]. By leveraging genetic variations that mimic the effects of modifiable exposures, MR allows for the dissection of causal pathways in complex diseases like breast cancer, circumventing confounding and reverse causation issues inherent in traditional observational studies [11].

In this study, we utilize a comprehensive two-sample MR analysis to investigate the causal impact of a wide array of immune cell features on breast cancer risk. This approach enables us to dissect the nuanced contributions of different immune cell types and states, providing a more detailed picture of the immune landscape in breast cancer [12]. Our findings hold potential implications for the development of novel immunotherapeutic strategies and could lead to more personalized approaches in breast cancer treatment and prevention. Through this research, we aim to add a significant layer of understanding to the immunological dimensions of breast cancer, addressing a critical gap in current cancer research. The results have the potential to redefine our approach to breast cancer management, moving towards more precise, immune-focused interventions. This study, therefore, holds substantial clinical significance, promising to influence future research directions, therapeutic strategies, and policy-making in the field of oncology.

## Materials and methods

### Study design

Based on two-sample MR Analysis, we assessed the causal relationship between 731 immune cell features (7 groups) and Breast cancer. MR uses genetic variation to represent risk factors, therefore instrumental variables in causal reasoning must satisfy Mendelian’s three major hypotheses [10, 11]. Ultimately, we evaluated the results by selecting instrumental variables, integrating exposure and outcome data, conducting MR analysis, and testing the stability of the results. Breast cancer dataset from IEU OpenGWAS database. The study examined GWAS in 257,730 Europeans, with 17,389 cases and 240,341 controls. The GWAS included 24,133,589 single nucleotide polymorphisms (SNPs) [13]. This is a study on a cross population map of 220 human phenotypic genetic associations, which identifies potential genetic components and identifies relevant variations and biological mechanisms in the current disease classification in the population.

### Immunity-wide GWAS data sources

Aggregate GWAS statistics for each immune trait are publicly available from the openGWAS (registration numbers from ebi-b-GCST90001391 to ebi-b-GCST90002121) [14]. A total of 731 immunophenotypes were analyzed, which comprised absolute cell counts (AC) (n = 118), median fluorescence intensity (MFI) representing surface antigen levels (n = 389), morphological parameters (MP) (n = 32), and relative cell counts (RC) (n = 192). Specifically, MFI, AC, and RC features include B cells, classic dendritic cells (cDCs), mature T cells, monocytes, bone marrow cells, T + B + NK (TBNK) cells, and regulatory T (Treg) cells, whereas MP features encompass CDC and TBNK cells [15]. The initial genome-wide association study (GWAS) for immune characterization utilized data from 3,757 Europeans, with no cohort overlap. SNPs for around 22 million high-density array genotypes were calculated using a Sardinian sequence-based reference panel, and correlations were investigated after adjusting for covariates such as sex and age [16].

### Selection of instrumental variables (IVs)

Since genetic variation is directly related to exposure, the significance level of IVs for each immune trait was set at 1×10^−5^. And all included SNPs are strongly correlated with immune cells (F>10, S1 Table).To obtain IVs for independent sites, we used the "Two Sample MR" packet data with a linkage unbalance (LD) threshold set to R^2^<0.001 and an aggregation distance of 10,000 kb [17]. For Breast cancer, we adjusted the significance level to 5×10^−8^, which is commonly used to represent genome-wide significance in GWAS, with a LD threshold of R^2^<0.001 and an aggregation distance of 10,000 kb [18].

### Statistical analysis

**Fig 1:**
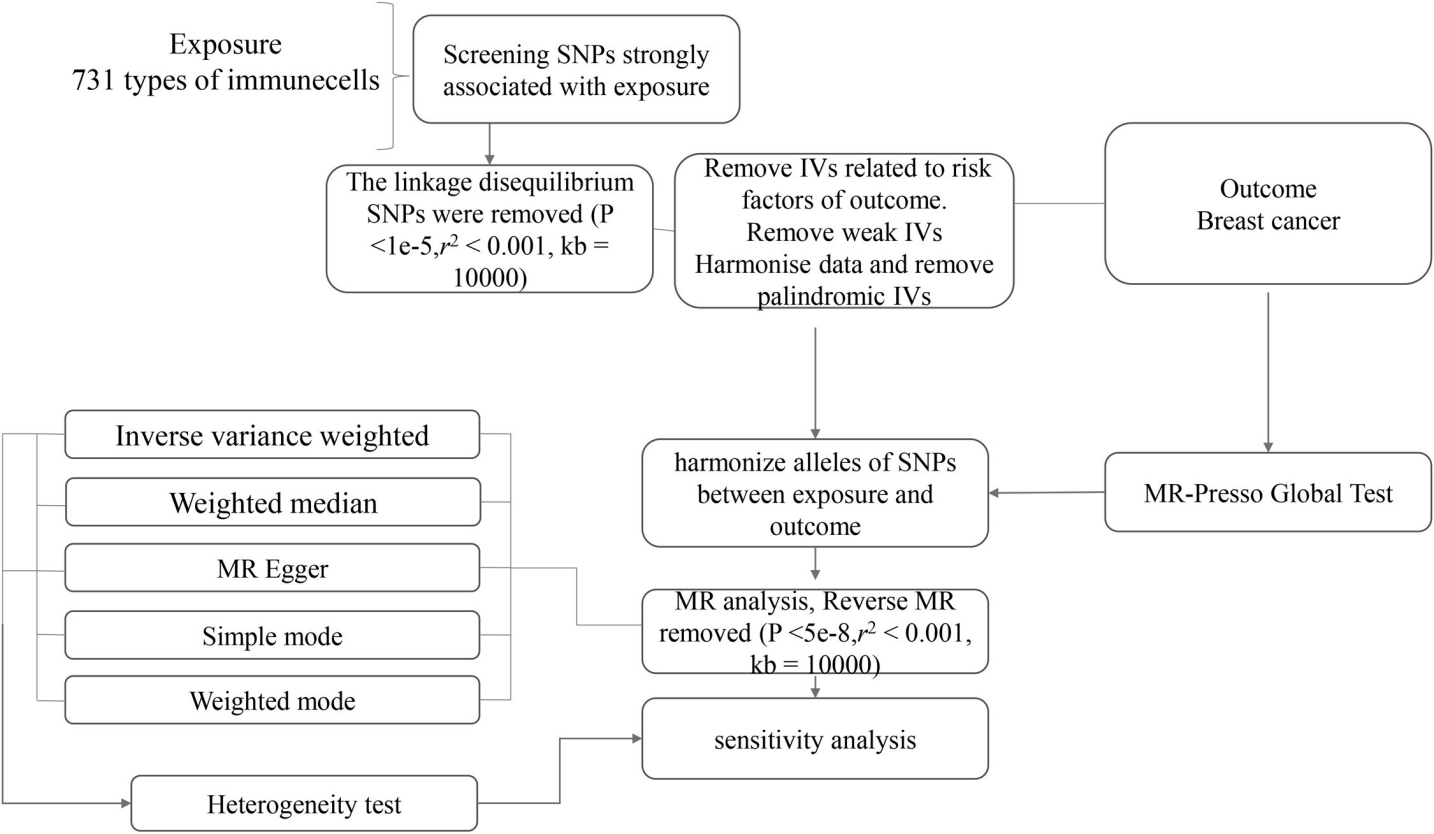
Flow diagram for quality control of the instrumental variables (IVs) and the entire Mendelian Randomization (MR) analysis process. *Abbreviations: SNPs, single-nucleotide polymorphisms; IVW, inverse variance weighted; MR, Mendelian Randomization; MR Presso, Mendelian Randomization Pleiotropy RESidual Sum and Outlier.

Mendelian randomization (MR) was used to investigate the causal relationship between 731 immune cells and breast cancer. Select single nucleotide polymorphisms (SNPs) significantly associated with the exposure of interest as instrumental variables (IVs). These SNPs were identified from genome-wide association studies (GWAS). For MR analysis, the inverse variance weighted (IVW) method is mainly used to estimate causal effects [19–22]. Supplementary analysis was conducted using methods such as MR Egger regression, weighted median, and weighted mode. Cochran’s Q-test was used to evaluate the heterogeneity between SNPs [23], and Bonferroni correction method was used to correct the results. In addition, a retention analysis was conducted to determine the impact of individual SNPs on the overall estimation [24]. All statistical analyses are conducted using R (4.3.2) [25]. The whole process was shown in **Fig 1**. The calculation method for F value is F = (beta/se) ^ 2.

## Results

### Exploration of the causal effect of immunophenotypes on Breast cancer risk

**Fig 2:**
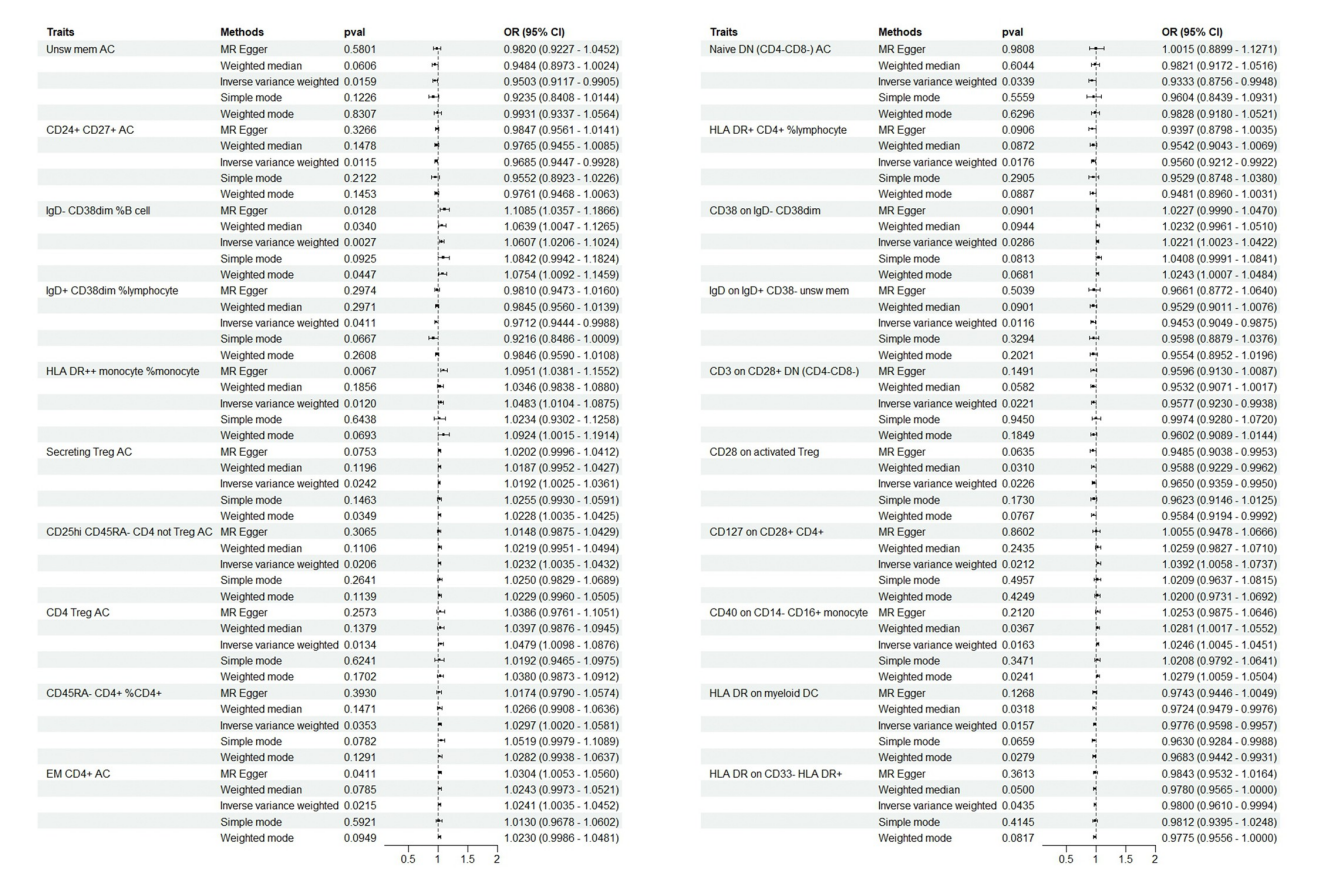
Forest plots depicting the causal associations between Breast cancer and specific immune cell traits. *Abbreviations: IVW, inverse variance weighting; CI, confidence interval.

At the significance level of 0.05, a total of 20 immunophenotypes were identified as causally associated with the development of Breast cancer. There were 6 cases in B cell group, 3 cases in Maturation stages of T cell group, 2 cases in TBNK cell group, 6 cases in Treg cell group, 1 case in cDC cell group, 1 cases in Monocyte group, and 1 cases in Myeloid cell group (as shown in **Fig 2**).

In B cell group, Unswmem AC (P = 0.015, OR = 0.950, 95%CI = 0.911~0.990), CD24+CD27+AC (P = 0.011, OR = 0.968, 95%CI = 0.944~0.992), IgD+CD38^dim^%lymphocyte (P = 0.041, OR = 0.971, 95%CI = 0.944~0.998) had a negative causal relationship with breast cancer, CD38 on IgD-CD38^dim^ (P = 0.028, OR = 1.022, 95%CI = 1.002~1.042), IgD-CD38^dim^%B cell (P = 0.002, OR = 1.060, 95%CI = 1.020~1.102) and IgD on IgD+CD38-unswmem (P = 0.011, OR = 0.945, 95%CI = 0.904~0.987) were positively correlated with breast cancer. In Maturation stages of T cell group, CD45RA-CD4+%CD4+(P = 0.035, OR = 1.029, 95%CI = 1.002~1.058) and EM CD4+AC (P = 0.021, OR = 1.024, 95%CI = 1.003~1.045) were positively correlated with breast cancer. Naive DN (CD4-CD8-) AC (P = 0.033, OR = 0.933, 95%CI = 0.875~0.944) and had a negative causal relationship with breast cancer. In TBNK cell group, HLA DR+ monocyte %monocyte (P = 0.011, OR = 1.048, 95%CI = 1.010~1.087) were positively correlated with breast cancer. HLA DR+CD4+%lymphocyte (P = 0.017, OR = 0.956, 95%CI = 0.921~0.992) had a negative causal relationship with breast cancer. In Treg cell group, CD127 on CD28+CD4+(P = 0.021, OR = 1.039, 95%CI = 1.005~1.073), Secreting Treg AC (P = 0.024, OR = 1.019, 95%CI = 1.002~1.036), CD25hi CD45RA-CD4 not Treg AC (P = 0.020, OR = 1.023, 95%CI = 1.003~1.043) and CD4 Treg AC (P = 0.013, OR = 1.047, 95%CI = 1.009~1.087) were positively correlated with breast cancer. CD3 on CD28+ DN (CD4-CD8-) (P = 0.022, OR = 0.957, 95%CI = 0.922~0.993) and CD28 on activated Treg (P = 0.022, OR = 0.964, 95%CI = 0.935~0.995) had a negative causal relationship with breast cancer. In cDC cell group, HLA DR on myeloid DC (P = 0.015, OR = 0.977, 95%CI = 0.959~0.995) had a negative causal relationship with breast cancer. In Monocyte group, CD40 on CD14-CD16+ monocyte (P = 0.016, OR = 1.024, 95%CI = 1.004~1.045) and were positively correlated with breast cancer. In Myeloid cell group, HLA DR on CD33-HLA DR+(P = 0.043, OR = 0.979, 95%CI = 0.960~0.999) had a negative causal relationship with breast cancer. Results from sensitivity analyses demonstrate the robustness of the observed causal association (**S1 Fig**). Scatter plot and funnel plot also show the stability of the results (**S2 and S3 Figs**).

### Exploration of the causal effect of Breast cancer risk on immunophenotypes

In order to study the causal relationship between Breast cancer and immunophenotype, two-sample MR Analysis was used, IVW method was the main analysis method, and other methods were auxiliary. We then used reverse MR To study the effect of Breast cancer onset on immunophenotypic immune cells. The results showed that there was no significant causal relationship between breast cancer and the above 20 immune characteristics.

### Stability of results

In our study, we used Cochran Q-test, MR Egger regression, and MR-PRESSO test to evaluate the stability of the results, and ultimately found no heterogeneity, outliers, or level pleiotropy (S2 and S3 Tables). We also used the Bonferroni correction method to correct the results, so the result with pval<0.05/731 = 0.000068 was considered significant, while the result with pval<0.05 was indicative. In this study, we screened confounding factors, but found no association between SNPs and breast cancer confounding factors, such as radiation, age.

## Discussion

The results of our study provide compelling evidence for the intricate relationship between various immune cell phenotypes and breast cancer risk, offering new insights into the immunological underpinnings of this disease. The identification of specific immunophenotypes with causal links to breast cancer not only enhances our understanding of the disease’s etiology but also opens avenues for novel therapeutic strategies [26]. Our analysis revealed a complex interplay between different immune cell types and breast cancer. While our study identified contain B cell phenotypes negatively associated with breast cancer, the mechanisms underlying this protective role merit further exploration. It is possible that these B cell subtypes contribute to effective anti-tumor immunity by presenting tumor antigens and facilitating the activation of T cells [27]. Conversely, other B cell subtypes might support tumor growth by creating an immunosuppressive environment or producing growth factors that aid tumor cells [28, 29]. Similarly, the positive correlation of contain Treg cell phenotypes with breast cancer risk raises questions about their exact role in tumor progression [30]. Treg cells are known to suppress immune responses, which in the context of cancer, could aid tumor evasion from immune surveillance [31]. Understanding the balance between their role in maintaining immune homeostasis and promoting tumor growth is crucial for developing targeted therapies. These findings are particularly relevant given the emerging role of these cell types in cancer immunotherapy. The positive and negative correlations observed among various immune phenotypes with breast cancer suggest a nuanced role of the immune system in tumor biology, which could be leveraged for therapeutic purposes [32, 33]. For instance, enhancing the activity of immune phenotypes negatively associated with breast cancer might offer a protective effect, whereas inhibiting those positively associated could reduce breast cancer risk [34].

The comprehensive analysis of immune cell phenotypes in relation to breast cancer risk creates an invaluable framework for advancing the field of immunotherapy. Despite the promising nature of current immunotherapeutic strategies, such as immune checkpoint inhibitors, their effectiveness in breast cancer has been notably limited. This limitation underscores a crucial need for the development of more refined and effective immunotherapeutic approaches that are specifically tailored to the unique immune landscapes characteristic of breast cancer patients [35].

Our findings provide pivotal insights that could significantly inform the development of these new strategies. By understanding the specific interactions between different immune cell phenotypes and breast cancer, we can better identify potential targets for therapy. This is especially crucial given the diverse roles that immune cells can play in cancer progression, ranging from tumor suppression to promotion. The ability to distinguish between these varying roles opens the door to therapies that can enhance the tumor-suppressing functions of the immune system while mitigating its tumor-promoting aspects. The diversity observed in the associations between immune cell phenotypes and breast cancer also highlights the burgeoning field of personalized medicine in oncology [36]. Recognizing and understanding the individual variations in immune cell phenotypes among breast cancer patients is key to developing more personalized and effective approaches for both prevention and treatment. This could manifest in several ways, such as the development of immune phenotype-based screening tools, which would allow for earlier and more accurate identification of breast cancer risk [28]. Furthermore, tailored immunotherapies that specifically target the unique immune components implicated in an individual’s cancer could offer more effective treatment options, reducing the risk of side effects and improving overall outcomes. Our study highlights several areas for future research. Further investigation into the mechanisms underlying the identified associations between immune phenotypes and breast cancer risk is warranted. Additionally, exploring the potential for combining immunophenotypic analysis with other biomarkers in breast cancer could enhance diagnostic and therapeutic precision [37]. The role of environmental and lifestyle factors in modulating these immune phenotypes also warrants further exploration [38]. While our study provides valuable insights, it is not without limitations. The use of two-sample MR analysis, although robust, relies on several assumptions and is subject to potential biases such as pleiotropy [39]. Furthermore, our study primarily focused on European populations, and the findings may not be generalizable to other ethnic groups [40]. Future studies should aim to include more diverse populations to ensure the applicability of the findings across different demographic groups.

In conclusion, our study elucidates the complex relationship between immune cell phenotypes and breast cancer risk, providing novel insights with significant clinical implications. The findings pave the way for more personalized and effective immunotherapeutic strategies in breast cancer treatment, emphasizing the importance of the immune system in the pathogenesis of this disease. As we advance our understanding of these relationships, we move closer to realizing the full potential of immunotherapy in breast cancer management.

## Supporting information

S1 Fig (A) B cell group sensitivity analyses plot. (B) Myeloid cell group sensitivity analyses plot. (C) T cell group sensitivity analyses plot. (D) TBNK cell group sensitivity analyses plot. (E) Treg cell group sensitivity analyses plot. (F) cDC cell group sensitivity analyses plot. (G) Monocyte group sensitivity analyses plot.
(ZIP)

S2 Fig (A) B cell group funnel plot. (B) Myeloid cell group funnel plot. (C) T cell group funnel plot. (D) TBNK cell group funnel plot. (E) Treg cell group funnel plot. (F) cDC cell group funnel plot. (G) Monocyte group funnel plot.
(ZIP)

S3 Fig (A) B cell group scatter plot. (B) Myeloid cell group scatter plot. (C) T cell group scatter plot. (D) TBNK cell group scatter plot. (E) Treg cell group scatter plot. (F) cDC cell group scatter plot. (G) Monocyte group scatter plot.
(ZIP)

S1 Table All SNPs strongly correlated with immune cells. (XLSX)

S2 Table Cochran Q-test and MR Egger regression to evaluate the stability of the results. (XLSX)

S3 Table MR-PRESSO test to evaluate the stability of the results. (XLSX)
